# Prediction of RNA Binding Residues: An Extensive Analysis Based on Structure and Function to Select the Best Predictor

**DOI:** 10.1371/journal.pone.0091140

**Published:** 2014-03-21

**Authors:** R. Nagarajan, M. Michael Gromiha

**Affiliations:** Department of Biotechnology, Indian Institute of Technology Madras, Chennai, India; Indian Institute of Science, India

## Abstract

Protein-RNA complexes play key roles in several cellular processes by the interactions of amino acids with RNA. To understand the recognition mechanism, it is important to identify the specific amino acids involved in RNA binding. Various computational methods have been developed for predicting RNA binding residues from protein sequence. However, their performances mainly depend on the training dataset, feature selection for developing a model and learning capacity of the model. Hence, it is important to reveal the correspondence between the performance of methods and properties of RNA-binding proteins (RBPs). In this work, we have collected all available RNA binding residues prediction methods and revealed their performances on unbiased, stringent and diverse datasets for RBPs with less than 25% sequence identity based on structural class, fold, superfamily, family, protein function, RNA type, RNA strand and RNA conformation. The best methods for each type of RBPs and the type of RBPs, which require further refinement in prediction, have been brought out. We also analyzed the performance of these methods for the disordered regions, structures which are not included in the training dataset and recently solved structures. The reliability of prediction is better than randomly choosing any method or combination of methods. This approach would be a valuable resource for biologists to choose the best method based on the type of RBPs for designing their experiments and the tool is freely accessible online at www.iitm.ac.in/bioinfo/RNA-protein/.

## Introduction

Protein-RNA interactions play significant roles in many biological processes such as mRNA stabilization and processing [1], protein synthesis [2], post translational modification [3], [4], assembly and function of ribosomes [5], eukaryotic spliceosomes assembly [6] and replication of virus [7], [8]. The specific interactions between amino acids and RNA provide valuable information to enhance our knowledge to understand the recognition mechanism of protein-RNA complexes. The binding sites in protein-RNA complex structures can be identified with atomic distance between the interacting residues in protein and RNA [9], solvent accessible area of bound and unbound protein [10] and energy based approaches [11]. Due to the experimental constraints in solving protein-RNA complex structures and the availability of large number of sequences [12], several methods have been proposed to identify the RNA binding sites from amino acid sequence using computational algorithms [13]–[24].

Wang and Brown (2006) proposed a Support vector machine (SVM) model trained with biochemical features of protein sequence and structure such as molecular mass, hydrophobicity, side chain pKa values, etc., for predicting the binding sites [15]. Further, they improved the prediction accuracy using evolutionary information in the form of position specific scoring matrices [22]. Kumar et al. (2008) utilized the composition of amino acids, residue pairs and PSSM profiles for identifying the binding sites [20]. Cheng et al. (2008) proposed a method based on smoothed PSSM along with dependency of neighboring residues [21]. NAPS [23] employed an ensemble based method using three algorithms such as C4.5, bootstrap aggregation and cost-sensitive learning to predict the binding sites. Ma et al. (2011) combined predicted secondary structure, polarity, hydrophobicity and evolutionary information for prediction [25]. Puton et al. (2012) developed a meta-predictor using the combination of three best performing methods, which outperforms all the three predictors [26]. Walia et al. (2012) reviewed the available prediction approaches and reported that the methods which use PSSM based sequence representation outperform smoothed PSSM and amino acid identity representation based methods [24]. Recently, Wang et al. (2013) utilized an extended naïve-Bayes-classifier for *de novo* prediction using protein and RNA sequence information [27]. The average accuracies of these methods are reported to be 70% to 80%. However the accuracy depends on the type of the RBP, features and algorithm. For example, the best performing method in one class/fold is poor in another type of class/fold (see below). Hence, it is important to reveal the correspondence between the type of a protein and performance of prediction methods.

In this work, we have classified the protein-RNA complexes into three broader categories based on (i) protein structure, (ii) RNA structure and (iii) protein function. The proteins belonging to these categories are further grouped into subclasses such as fold, superfamiliy and family based on protein structure; RNA conformation, strand and type based on RNA structure; protein functions such as enzymes, regulatory proteins and structural proteins. On the other hand, we have collected all the available prediction methods, which have either on-line tool or standalone program for identifying the RNA binding residues. We have developed necessary in-house programs for analyzing the performance of all the available methods in all the data sets. We have related the performance of each method with different datasets and revealed the correspondence between them. The results obtained from our analysis would be helpful to the researchers to choose the best method for their protein(s) of interest to design experiments and opens up the necessity of new/refinement of methods for certain type of RBPs. Further, the performance of methods in specific subtypes of RBPs will be discussed.

## Materials and Methods

### Data sets

We have collected all the protein-RNA complexes (1472 entries) available in Protein Data Bank (PDB) [30] (last accessed on 17^th^ July 2013) and classified them into three major categories based on (i) protein structure, (ii) RNA structure and (iii) protein function. We followed the classification of SCOP [31] for categorizing them into different classes, folds, superfamilies and families. The RBPs in these categories have been culled with the sequence identity of less than 25% to avoid the bias in the analysis. Final dataset contains 172 protein chains from 8 classes, 90 folds, 100 superfamilies and 126 families.

RNA structures are classified on three aspects: (i) RNA conformation such as A, RH, T and U, (ii) strand of RNA (single stranded and double helical) and (iii) type of RNA. The structural details of RNA have been obtained from Nucleic acid database (NDB) [32]. The final dataset contains 185, 186 and 110 protein chains with the sequence identity of less than 25% based on RNA conformation, strand and type, respectively.

The functional classification is based on enzymes, regulatory proteins and structural proteins, which are obtained from NDB. The final dataset contains 64 enzymes, 23 regulatory proteins and 76 structural proteins with the sequence identity of less than 25%.

### Computational methods for RNA binding residues prediction

We have collected all the available methods for predicting the binding sites in RBPs from amino acid sequence, which have either online services or available standalone program. The methods are BindN [15], Pprint [20], RNAProB [21], BindN+ [22], NAPS [23] and RNABindR v2.0 [24]. The details regarding name, features, technique, reference and link for the methods used in the present work are listed in Table **S1**. These methods used different datasets and their reported accuracies lie in the range of 70–80%.

### Identification of RNA interacting residues

In this approach, we used two different distance based criteria to identify the RNA binding residues to analyze the performance of prediction methods. A residue in a RBP is identified as binding if the distance between any of its heavy atoms and a heavy atom in RNA is ≤3.5 Å (or ≤6.0 Å). Based on the distance criteria, we have developed in-house programs for identifying RNA binding residues for all the protein-RNA complexes in all the datasets. Generally, distances in the range of 3.5 Å to 6.0 Å is used in the literature and most of the prediction methods used the cutoff of 3.5 Å for identifying the binding site residues [15], [22], [23]. It is advised to use ≤3.5 Å for stringent prediction and ≤6.0 Å for flexible prediction in consideration with the experimental noise.

### Assessing the performance of prediction methods

We have assessed the performance of different methods using various measures such as sensitivity, specificity, accuracy and Matthews correlation coefficient (MCC). Sensitivity depicts the correct prediction of RNA binding residues, specificity reveals the ability of excluding non-binding residues and accuracy provides the overall performance. Accuracy2 (or balanced accuracy) is the mean of sensitivity and specificity, which avoids overestimating the prediction performance of methods on imbalanced datasets. Hence, we used Accuracy2 to select the best method in all the classifications. We also considered cutoff value of >60% for both sensitivity and specificity. Other measures accuracy and MCC were treated with less priority.
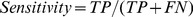
(1)

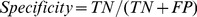
(2)

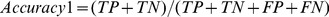
(3)

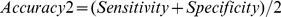
(4)

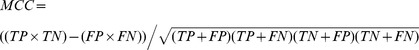
(5)


In these equations, TP is the number of true positives (binding residues predicted as binding), TN is the number of true negatives (non-binding residues predicted as non binding), FP is the number of false positives (non-binding residues predicted as binding) and FN is the number of false negatives (binding residues predicted as non binding).

## Results and Discussions

### Structural classes

Protein-RNA complexes are classified into 8 classes such as all-α, all-β, α+β, α/β, multi domain proteins, small proteins, peptides and low resolution structures. The prediction performances of all the methods in these classes are shown in the Table **1**. The methods BindN+ and Pprint showed the best performance in most of the classes for ≤3.5 Å and ≤6.0 Å distance criteria, respectively. RNABindR v2.0 performed well with the accuracy of more than 70% in six of the eight classes using the cutoff distance of 3.5 Å. However, the accuracy is less than 60% in small proteins. The method NAPS has the accuracy of less than 60% in six of the eight structural classes using the cutoff distance of 6.0 Å.

**Table 1:** Prediction accuracy (%) of binding sites in different structural classes.

Method	Average	all-α	all-β	α+β	α/β	Low resolution	Multidomain	Peptides	Small proteins
**Distance cutoff ≤3.5 Å**									
BindN	66.14	62.74	63.52	62.80	64.91	72.11	65.26	66.66	71.08
BindN+	75.71	75.37	75.50	73.58	75.20	81.64	74.59	76.81	74.77
NAPS	64.46	63.12	60.39	60.64	63.19	64.67	60.22	82.97	60.45
Pprint	72.70	70.40	71.12	70.13	73.10	70.55	70.10	84.78	71.35
RNABindR v2.0	72.00	75.40	67.22	70.93	73.25	70.76	73.74	89.13	55.63
RNAProB	66.53	66.05	70.13	65.61	71.58	71.69	61.43	68.12	57.59
**Distance cutoff ≤6.0 Å**									
BindN	60.99	59.68	59.95	59.62	60.95	69.07	59.50	57.69	61.49
BindN+	69.95	69.35	69.70	67.90	67.69	79.11	64.38	69.95	71.50
NAPS	60.12	59.72	57.33	57.96	58.34	60.62	57.27	70.67	59.03
Pprint	73.46	70.71	71.23	69.89	73.51	76.90	68.04	79.09	78.28
RNABindR v2.0	69.51	73.33	66.95	68.55	70.59	72.50	67.81	78.37	57.98
RNAProB	64.54	62.52	64.88	63.36	65.06	69.39	56.97	78.37	55.76

Accuracy  =  (sensitivity+specificity)/2.

Interestingly, the performance of different methods varies with cutoff distance. With 3.5 Å distance cutoff, BindN+ uniformly performs well in all the classes. On the other hand, with 6.0 Å distance cutoff the best prediction method depends on the class. For example, RNABindR v2.0 showed the highest accuracy in all-α class whereas Pprint has the best performance in all-β class proteins.

### Folds, superfamilies and families

**Figure 1:**
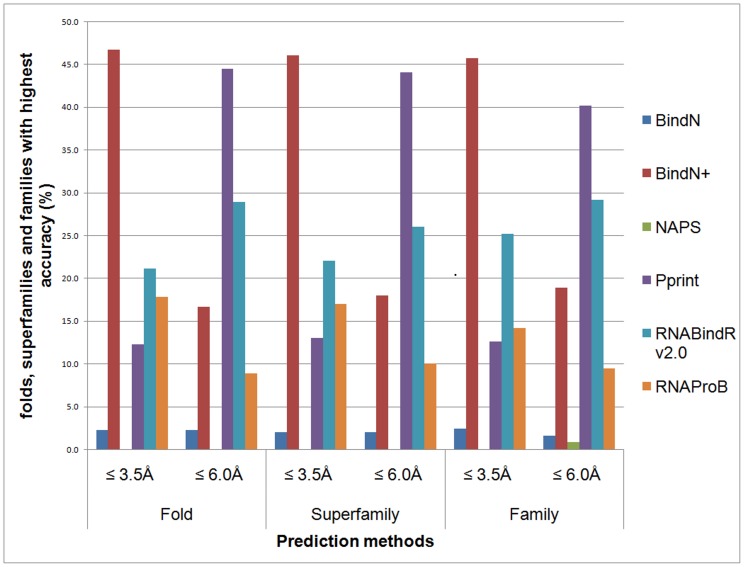
Prediction performance of computational methods in various folds, superfamilies and families.

We have analyzed the prediction performance of all the computational methods in 90 folds, 100 superfamilies and 126 families and the results are presented in the Figure 1. In all the three classifications (folds, superfamilies and families), BindN+ and Pprint showed the best performance in >40% of folds, superfamilies and families using the distance cutoff of 3.5 Å and 6.0 Å, respectively. In addition, other methods also performed well in few folds, superfamilies and families (Figure 1). These results showed the importance of different methods with different levels of performance.

Further, we have identified methods, which have the best and least performance in each fold, superfamiliy and family and the results are represented in Table **S2 in Tables S1**. Table **2** lists few typical examples from each category. BindN+ and Pprint showed the best performance in the fold type of NSP3 homodimer and ribosomal proteins with an accuracy of 91% and 90%, respectively. However, methods NAPS and RNAProB showed a poor performance with accuracy of about 60–61%. We also presented two examples of poorly predicted folds in Table **2**. The best accuracy of Nucleoplasmin-like fold is just 63% with the sensitivity and specificity of 31% and 95%, respectively.

**Table 2:** Typical examples of best and least predicted folds, superfamilies and families.

Fold/superfamily/family	Distance (Å)	Best Method	Sensitivity	Specificity	Accuracy1	Accuracy2	MCC	LowestAccuracy2	Method	MCC
**Fold**										
NSP3 homodimer (1)	≤ 3.5	BindN+	93.75	89.86	90.26	91.81	0.65	60.28	NAPS	0.14
Ribosomal proteins (2)	≤ 6.0	Pprint	90.62	88.73	91.40	89.68	0.79	61.60	RNAProB	0.32
*Nucleoplasmin (3)*	≤ 3.5	*RNAProB*	*31.11*	*94.79*	*94.09*	*62.95*	*0.22*	*43.81*	*RNABindR v2.0*	*0*
*IF3-like (1)*	≤ 6.0	*Pprint*	*38.46*	*84.13*	*82.88*	*61.30*	*0.10*	*47.91*	*BindN*	*0*
**Superfamily**										
Rho N-terminal domain(1)	≤ 3.5	RNAProB	100.00	97.25	97.46	98.62	0.85	49.70	NAPS	0
tRNA-binding arm (1)	≤ 6.0	RNAProB	88.89	99.72	99.46	94.31	0.89	61.39	NAPS	0.07
*proRS (1)*	≤ 3.5	*BindN+*	*44.44*	*91.59*	*90.70*	*68.02*	*0.17*	*48.60*	*RNAProB*	*0*
*Poly A polymerase (1)*	≤ 6.0	*RNABindR v2.0*	*42.86*	*78.82*	*76.61*	*60.84*	*0.12*	*47.87*	*BindN+*	*0*
**Family**										
SM motif of SNRNP (1)	≤ 3.5	RNAProB	83.33	93.94	93.06	88.63	0.65	43.94	BindN+	0
L23p(1)	≤ 6.0	Pprint	88.57	95.65	92.59	92.11	0.85	62.86	RNAProB	0.41
*RNB domain (1)*	≤ 3.5	*RNABindR v2.0*	*61.76*	*70.61*	*70.17*	*66.19*	*0.15*	*48.02*	*RNAProB*	*0*
*Comoviridae (2)*	≤ 6.0	*NAPS*	*42.11*	*76.35*	*74.59*	*59.23*	*0.09*	*46.72*	*Pprint*	*0*

Accuracy1  =  (TP + TN)/(TP + TN + FP + FN).

Accuracy2  =  (sensitivity + specificity)/2.

Among the superfamilies, Rho N-terminal domain-like and tRNA-binding arm are predicted with the accuracy of >90% by RNAProB whereas the lowest accuracies are 50% and 61%, respectively. The proRS and poly A polymerase are the examples of poorly predicted superfamilies with the sensitivity of less than 45%. Although BindN+ performs well in many superfamilies, it is identified as the least accuracy method in poly A polymerase superfamily. The families SM motif of SNRNP and L23p are predicted with the highest accuracy of 89% and 92% whereas RNB domain-like and Comoviridae-like VP are poorly predicted with accuracy of 66% and 59%, respectively. These results showed that the prediction methods are complementing each other in different types of RBPs.

### Disordered regions

**Figure 2:**
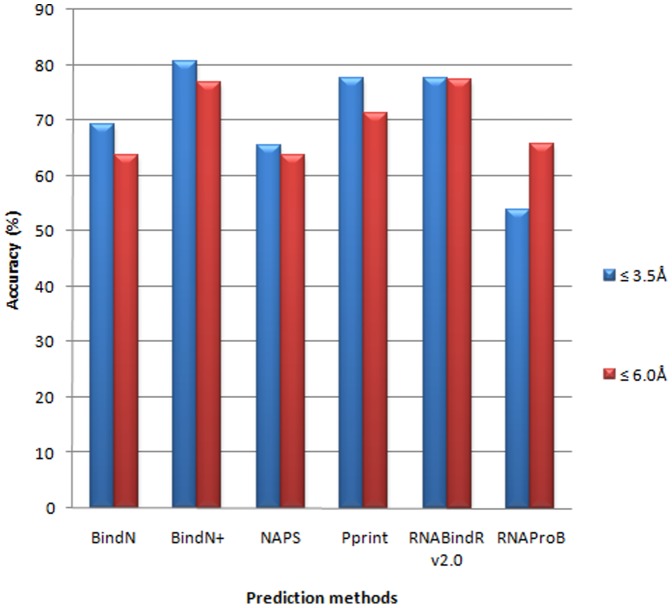
Prediction performance of computational methods in disordered regions.

We have collected the disordered regions by comparing the proteins in free and complex forms and identified the binding sites using the complex structures. These information have been used to evaluate the performance of different prediction methods in disordered regions. The results obtained in disordered regions of 33 protein chains are presented in Figure 2 and Table **S3 in Tables S1**. Interestingly, all the methods except RNAProB perform well with an average accuracy of more than 60%. Further, BindN+ showed an average accuracy of 81%, which is remarkably higher than that obtained in DNA binding proteins, which have the average accuracy of 65% [28], [29].

### RNA structure

We have classified protein-RNA complexes based on the types of RNA strand such as single strand and double helical RNA. We observed that RNABindR v2.0 has the best performance in double helical RNA. In single stranded RNA, BindN+ and Pprint predicted well in ≤3.5 Å and ≤6.0 Å distance, respectively. The methods RNAProB and NAPS have poor performance with the accuracy of less than 60% in double helical RNA.

We have grouped the protein-RNA complexes into 8 different groups based on the type of RNA. The best and least accuracy method in all the RNA types is shown in Table **3** and Table **S4 in Tables S1**. We noticed that RNABindR v2.0 has the best performance in mRNA and siRNA with an accuracy of about 79% and 80%. In the rRNA type, BindN+ and Pprint are showed the best performance at the cutoff distance of 3.5 Å and 6.0 Å, respectively. The accuracy is more than 75% with a balance between sensitivity and specificity. This might be due to the fact that rRNA type has more number of structures and are well trained for prediction. Viral RNA has 12 proteins and the highest accuracy in this type is 66% with the low sensitivity of 49%.

**Table 3:** Prediction accuracy of binding sites in different RNA types (3.5 Å cutoff).

RNA type	Best Method	Sensitivity	Specificity	Accuracy1	Accuracy2	MCC	Lowest Accuracy2	Method	MCC
mRNA (7)	RNABindR v2.0	77.03	80.48	80.48	78.76	0.35	56.74	NAPS	0.07
Pre miRNA(2)	BindN	80.00	81.38	81.20	80.69	0.19	45.70	RNAProB	0
rRNA(54)	BindN+	83.01	74.65	79.08	78.83	0.52	63.62	NAPS	0.24
sRNA(1)	RNAProB	61.90	96.81	90.43	79.36	0.66	60.81	BindN	0.23
siRNA(3)	RNABindR v2.0	82.64	77.89	79.82	80.27	0.48	60.93	Pprint	0.19
snRNA(3)	BindN+	90.11	91.15	90.65	90.63	0.66	73.64	NAPS	0.20
tRNA(28)	RNAProB	59.86	93.74	91.49	76.80	0.44	59.25	NAPS	0.09
viral_RNA(12)	BindN+	49.21	83.38	81.90	66.29	0.17	57.60	NAPS	0.07

Accuracy1  =  (TP + TN)/(TP + TN + FP + FN).

Accuracy2  =  (sensitivity + specificity)/2.

The best performance of prediction methods in each RNA conformation is shown in Table **S5 in Tables S1**. Most of the RNA structures have the conformation type of U-type and less structures are available with RH-type. The method RNABindR v2.0 showed the best performance in A-type and RH-type. In U-type, BindN+ and Pprint showed the highest accuracy of 72% and 69% at the distance cutoff of 3.5 Å and 6.0 Å, respectively.

### Protein function

Based on protein functions, we have grouped the protein-RNA complex structures into three major categories such as enzymes, regulatory and structural proteins and the prediction results are presented in Tables **S6-S8 in Tables S1**. The structures in the enzyme group are further divided into 9 groups with 58 chains. The binding sites of most of the enzymes are well predicted with an accuracy of more than 70% using BindN+, RNABindR v2.0 and Pprint. We observed similar results in regulatory proteins. In structural proteins, RNAProB predicted well in two of the four cases.

### Performance of prediction methods in different datasets

We have evaluated the performance of methods using two different and independent datasets: i) dataset of structures, which are not included in the training dataset for developing individual prediction methods and ii) dataset of recently solved protein-RNA complex structures (since June 2012). The prediction results of each method in these two datasets were presented in the Table **4**. The accuracy of all the methods in both datasets lies between 55–71%. BindN+ and RNABindR v2.0 showed the best performance in both datasets at the distance cutoff of ≤3.5 Å and ≤6.0 Å, respectively. Pprint and NAPS performed well with an average accuracy of 66% and 61%, respectively. The performance of the method RNAProB is poor for the newly crystallized protein-RNA complex structures with the average accuracy of 55%.

**Table 4:** Prediction performance of different methods in two independent datasets.

Method	Data set 1						Data set 2					
	≤3.5 Å			≤6.0 Å			≤3.5 Å			≤6.0 Å		
	Accuracy1	Accuracy2	MCC	Accuracy1	Accuracy2	MCC	Accuracy1	Accuracy2	MCC	Accuracy1	Accuracy2	MCC
BindN	74.88	64.00	0.23	70.35	61.26	0.21	75.49	62.78	0.21	71.26	60.98	0.20
BindN+	79.45	70.65	0.34	77.43	66.93	0.33	78.75	68.01	0.30	76.77	65.46	0.30
NAPS	66.29	60.89	0.17	63.58	58.36	0.15	66.61	62.80	0.18	64.55	60.61	0.16
Pprint	70.74	66.22	0.25	73.82	66.59	0.31	70.70	64.80	0.21	72.05	65.17	0.26
RNABindR v2.0	65.95	68.78	0.27	71.28	67.38	0.32	65.12	66.90	0.22	70.47	66.72	0.28
RNAProB	82.21	60.15	0.22	73.43	58.47	0.21	80.48	55.71	0.13	71.03	55.20	0.13

Data set 1: List of protein-RNA complexes analyzed in this work and are not used in the respective methods.

Data set 2: List of protein-RNA complexes published since June 2012, after the publication of analyzed prediction methods.

We have classified the prediction methods into three groups such as i) additive ii) PSSM iii) non-PSSM. The performances of these three groups in all our datasets are given in Table **S9 in Tables S1**. From the results, it is clearly seen that the PSSM group performs very well in all the 13 datasets with the accuracy of more than 60%. This trend is similar to the prediction of DNA binding residues [28] and other reports in the literature [24].

### Comparison between ensemble method and best methods

We have performed an ensemble based prediction, which is based on the majority of voting among six methods used in this work. We have compared the ensemble based prediction results with the best methods in all the datasets and the results are shown in the Table **5**. Interestingly, the best methods identified in each classification outperformed the ensemble based prediction in most of the datasets. For example, ensemble based prediction has the highest accuracy in 17 out of 126 families whereas it is 109 using the best methods identified in this work. The average accuracy has been improved up to 8% using the best method. Further, the comparison of prediction accuracies in all the 290 RNA binding protein chains showed that 68% and 57% of the complexes has been better predicted with the accuracy of at least 5% using the method identified in this work than the ensemble based method at the cutoff of 6 Å and 3.5 Å, respectively. The comparison demonstrated the importance of using the best method to predict RNA binding residues.

**Table 5:** Comparison between ensemble method and best methods in different datasets.

Data set	Number of sub groups	Number of sub groups predicted with highest accuracy				
		≤3.5 Å		≤6.0 Å		
		Ensemble	Best method	Ensemble	Best method	
Class	8	2 (76.03)	6 (77.48)	0 (69.69)	8 (74.06)	
Fold	90	11 (74.09)	79 (80.93)	4 (68.69)	86 (76.87)	
Superfamily	100	13 (74.08)	87 (80.94)	4 (68.68)	96 (76.85)	
Family	126	17 (73.67)	109 (80.67)	5 (68.15)	121 (76.63)	
RNA conformation	4	0 (70.69)	4 (75.23)	0 (65.35)	4 (71.22)	
RNA strand	2	0 (67.48)	2 (71.31)	0 (63.57)	2 (68.56)	
RNA type	8	0 (70.61)	8 (78.95)	0 (64.48)	8 (72.48)	
Protein function	21	1 (68.11)	20 (75.74)	0 (62.48)	21 (70.79)	

Average accuracies (%) are given in parentheses.

### Web application

**Figure 3:**
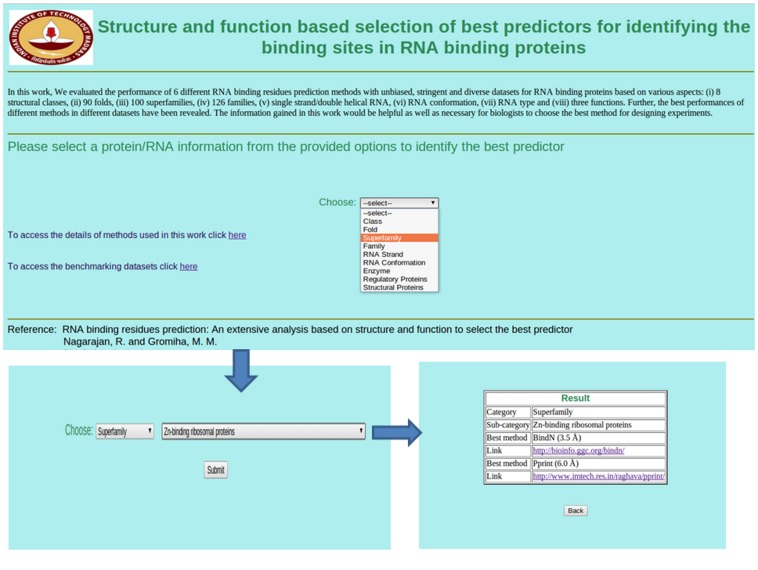
Web application to provide the best methods based on the type of RBPs.

We have developed a user friendly web application using PERL-CGI modules for back end and HTML and JavaScript for front end. This application is designed to provide the best method for any RBP based on its structural class, fold, superfamily, family, RNA strand, RNA type, RNA conformation and protein function. It takes structural/functional information of a query RBP/RNA and displays the best method and corresponding link to access the method in the output. The web application is freely available and can be accessed at www.iitm.ac.in/bioinfo/RNA-protein/. For example, in the superfamily of Zn-binding ribosomal protein, BindN and Pprint are the best methods at the distance cutoff of ≤3.5 Å and ≤6.0 Å, respectively (Figure 3). We also included separate links for accessing all the datasets used in this work for evaluating the prediction methods and the list of analyzed methods with appropriate details.

## Conclusions

Computational methods provide consensus as well as conflict prediction results on identifying the RNA binding site residues. It is essential to reveal the best and reliable method for a query RBP. Based on the structure and function of the interacting protein and RNA, we have analyzed the available RNA binding sites prediction methods using stringent, unbiased and diverse data sets. We revealed the one-to-one correspondence between prediction performance of methods and the type of RBPs. We have also developed a web application to choose the best method for any RBP of interest. The results obtained in this work would aid biologists to design experiments efficiently. Secondly, the analysis pointed out the subgroup of RBPs, which requires new method or refinement of methods. Further, the performance of PSSM based methods are better than other features based on physio-chemical characteristics of amino acid residues.

## Supporting Information

Tables S1 Table S1. List of prediction methods used in the present study. Table S2. Performance of prediction methods in each fold, superfamily and family. Table S3. Prediction performance of methods in disordered regions. Table S4. Prediction performance of methods in different RNA types. Table S5 Prediction performance of methods in different RNA conformation. Table S6. Prediction performance of methods in each enzyme. Table S7. Prediction performance of methods in each regulatory protein. Table S8. Prediction performance of methods in each structural protein. Table S9. Prediction performance of three groups of methods in all the datasets.
(XLS)
